# Simultaneous emission and attenuation reconstruction in time-of-flight PET using a reference object

**DOI:** 10.1186/s40658-020-0272-9

**Published:** 2020-01-13

**Authors:** Pablo García-Pérez, Samuel España

**Affiliations:** 1 Departamento de Estructura de la Materia, Física Térmica y Electrónica, Facultad de Ciencias Físicas, Universidad Complutense de Madrid, IdISSC, Ciudad Universitaria, 28040 Madrid, Spain; 2 Centro Nacional de Investigaciones Cardiovasculares (CNIC), Madrid, Spain

**Keywords:** Positron emission tomography, Attenuation correction, Joint reconstruction, Time of flight

## Abstract

**Background:**

Simultaneous reconstruction of emission and attenuation images in time-of-flight (TOF) positron emission tomography (PET) does not provide a unique solution. In this study, we propose to solve this limitation by including additional information given by a reference object with known attenuation placed outside the patient. Different configurations of the reference object were studied including geometry, material composition, and activity, and an optimal configuration was defined. In addition, this configuration was tested for different timing resolutions and noise levels.

**Results:**

The proposed strategy was tested in 2D simulations obtained by forward projection of available PET/CT data and noise was included using Monte Carlo techniques. Obtained results suggest that the optimal configuration corresponds to a water cylinder inserted in the patient table and filled with activity. In that case, mean differences between reconstructed and true images were below 10%. However, better results can be obtained by increasing the activity of the reference object.

**Conclusion:**

This study shows promising results that might allow to obtain an accurate attenuation map from pure TOF-PET data without prior knowledge obtained from CT, MRI, or transmission scans.

## Background

In order to obtain quantitative images in positron emission tomography (PET), corrections such as attenuation, scatter, and randoms must be implemented. Attenuation information in early PET scanners was obtained using transmission sources, while attenuation maps are currently derived from computed tomography (CT) images on hybrid PET/CT scanners.

In combined PET/MRI (magnetic resonance imaging) scanners, MRI provides anatomic images with better soft tissue contrast than CT and no ionization radiation is used, reducing the dose delivered to the patient. In addition, other applications such as spectroscopy or molecular imaging are also possible with MRI [1, 2]. Furthermore, development of stand-alone PET scanners dedicated to specific anatomical regions like the brain [3, 4] or breast [5] has been pursued in the last decade motivated by the need of improved scanner performance. However, hybrid PET/MRI and stand-alone PET scanners lack accurate methods for attenuation correction. Therefore, simplified attenuation maps are derived from MR images [6–8] or when no transmission data is available [9–11]. A promising approach suggested the joint reconstruction of emission and attenuation images from time of flight (TOF)-PET data using algorithms like maximum likelihood estimation of attenuation and activity (MLAA) [12–15]. However, as pointed by Defrise et al. [16], a major drawback of this approach is that the attenuation sinogram can be only determined up to an additive constant. This limiting factor is considered to be the main reason why these methods are still not implemented in clinical practice [17].

Several methods have been proposed to overcome this limitation. Some of these methods rely on available CT data [17–20] which might not be available in most cases where the use of simultaneous reconstruction of emission and attenuation is valuable like PET/MRI and stand-alone PET scanners. Other methods propose searching for an optimal initialization of the attenuation map based on MR images [14, 20, 21], which are prone to segmentation and misclassification errors, are limited by data availability, and rely on patient databases. Other authors attempted to accomplish this task by adding transmission sources in the scanner [22, 23], using the lutetium background radiation source as the transmission source [24], or trying to use scattered events from emission data [25, 26].

In this work, we evaluated a strategy suggested by Defrise et al. [16] that uses a reference object external to the patient with known attenuation values to overcome the limitation of the additive constant. For that purpose, a modified MLAA reconstruction algorithm was proposed and different configurations of the reference object were evaluated.

## Materials and methods

### Reconstruction algorithm

MLAA algorithm is based on two iterative procedures. For the reconstruction of the activity distribution, a maximum likelihood expectation maximization (MLEM) procedure is used with the TOF-PET sinogram and the attenuation map as input. On the other hand, the reconstruction of the attenuation map is performed using a maximum likelihood gradient ascent procedure with the TOF-PET sinogram and the activity distribution as input. As both activity and attenuation images are unknown, these procedures are concatenated every iteration or few iterations leading to the mentioned simultaneous reconstruction of both images.

The MLEM algorithm used in this work can be expressed as:

1 $$ {\lambda}_j^{\left(n+1\right)}={\lambda}_j^{(n)}\bullet \frac{\sum_{i,\mathrm{TOF}}{c}_{ij}^{\mathrm{TOF}}\frac{y_{i,\mathrm{TOF}}}{b_{i,\mathrm{TOF}}^{(n)}}}{\sum_{i,\mathrm{TOF}}{c}_{ij}^{\mathrm{TOF}}{a}_i^{(n)}} $$

where ${\lambda}_j^{(n)}$ is the estimated activity at iteration *n* on image voxel *j*, *y*_*i*, TOF_ is the measured number of coincidences in sinogram entry (*i*,TOF), ${c}_{ij}^{\mathrm{TOF}}$ is the system matrix which reflects the sensitivity of pixel *j* with respect to sinogram entry (*i*,TOF), ${a}_i^{(n)}$ is the estimated attenuation sinogram, and ${b}_{i,\mathrm{TOF}}^{(n)}$ is the estimated emission sinogram. ${c}_{ij}^{\mathrm{TOF}}$ was estimated using a Siddon’s ray tracing algorithm [27] and the TOF resolution model was included with a Gaussian profile according to the employed coincidence resolving time (CRT). ${b}_{i,\mathrm{TOF}}^{(n)}$ and ${a}_i^{(n)}$ can be expressed as:

2 $$ {b}_{i,\mathrm{TOF}}^{(n)}={\sum}_j{c}_{ij}^{\mathrm{TOF}}{\lambda}_j^{(n)}\ {a}_i^{(n)}=\exp \left(-{\sum}_j{l}_{ij}{\mu}_j^{(n)}\right) $$

where ${\mu}_j^{(n)}$ is the estimated attenuation coefficient and *l*_*ij*_ is the intersection length of the line of response (LOR) for sinogram entry *i* with pixel *j*.

The maximum likelihood gradient ascent algorithm used for the reconstruction of the attenuation map can be expressed using the following equation:

3 $$ {\mu}_j^{\left(n+1\right)}={\mu}_j^{(n)}+\frac{\alpha_p}{D}\left(1-\frac{\sum_i{c}_{ij}{y}_i}{\sum_i{c}_{ij}\left({a}_i^{(n)}{b}_i^{(n)}\right)}\right) $$

where *α*_*p*_ is a relaxation coefficient and *D* the diameter of the PET ring [12, 14]. Note that no TOF information is used in this case. The results shown in this study were obtained after 1000 iterations updating the attenuation map according to Eq. 3 every 3 MLEM iterations (see Eq. 1) and with *α*_*p*_ = 2 and *D* = 903 mm.

As pointed out by Defrise et al. [16], the MLAA algorithm allows to calculate the attenuation sinogram up to an additive constant. They also mentioned that if the attenuation coefficient is known for some LORs, the emission data determine in a unique way all the attenuation factors. For that purpose, they proposed using a reference object with known attenuation and activity placed outside the convex hull of the scanned object to recover the attenuation factors for all LORs. This is possible because the attenuation is thus known for any LOR that crosses this reference object but does not cross the scanned object. However, to the best of our knowledge, this strategy has not been tested to date.

In order to implement this technique, the MLAA algorithm was modified as follows. Every time the attenuation map is updated by (3), an additive constant is added to the entire map forcing the pixels within the reference object to have an average attenuation coefficient according to its known value (${\overline{\mu}}_{\mathrm{ref}}$). For that purpose, the difference between ${\overline{\mu}}_{\mathrm{ref}}$ and the mean attenuation coefficient within a region of interest (ROI) drawn inside the reference object in the attenuation image at current iteration (${\overline{\mu}}_{\mathrm{ROI}}^{(n)}$) is obtained, and the entire attenuation map is corrected by this difference as follows:

4 $$ {\mu}_{j,\mathrm{corr}}^{(n)}={\mu}_j^{(n)}+{K}_{\mathrm{corr}}^{(n)} $$

where ${K}_{\mathrm{corr}}^{(n)}$ is given by

5 $$ {K}_{\mathrm{corr}}^{(n)}={\overline{\mu}}_{\mathrm{ref}}-{\overline{\mu}}_{\mathrm{ROI}}^{(n)} $$

In this way, ${\mu}_{j,\mathrm{corr}}^{(n)}$ is used as the attenuation map for the next iteration.

### 2D simulation

**Fig. 1:**
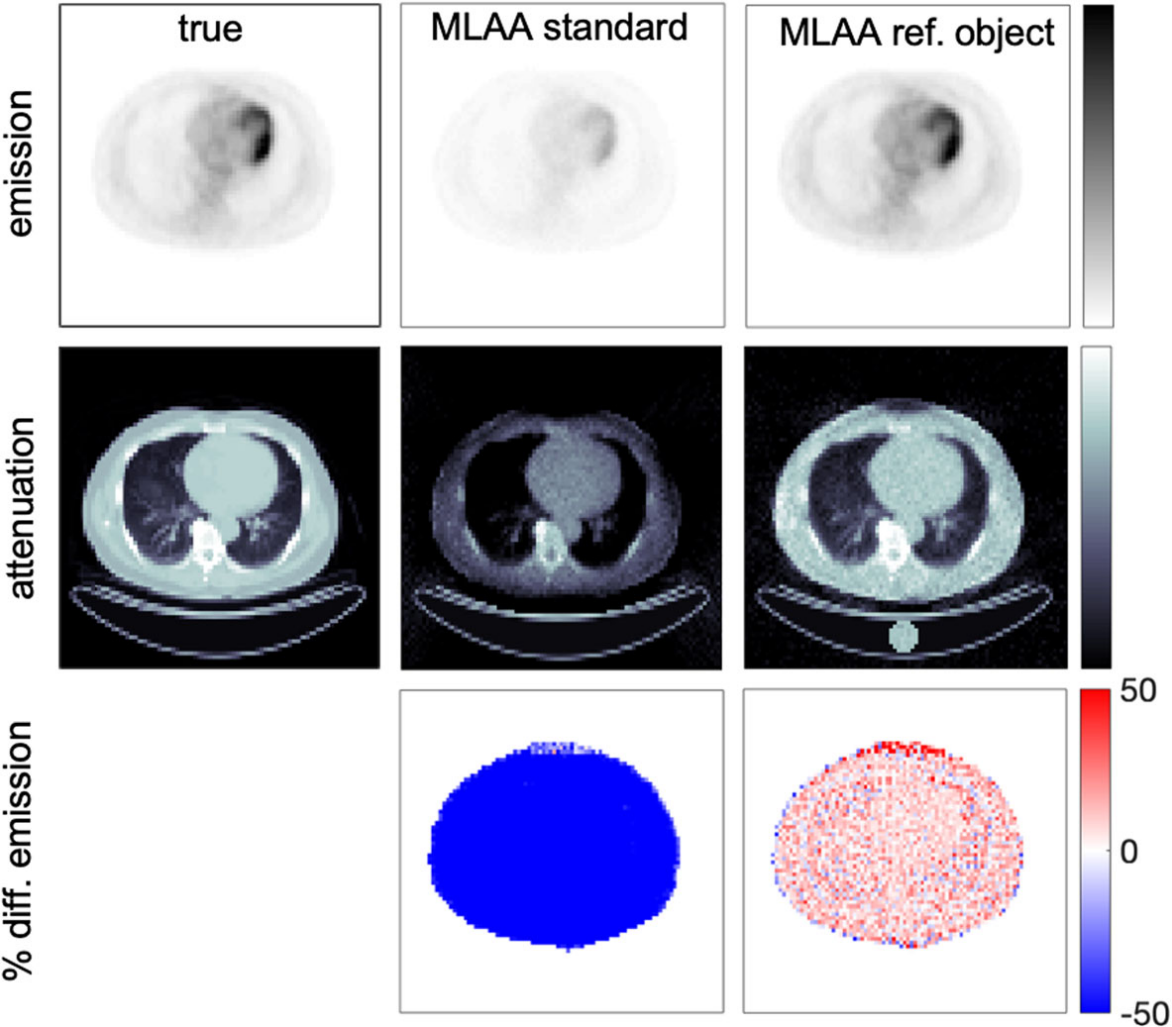
Reconstructed emission and attenuation images and percentage difference maps of emission images obtained with standard MLAA reconstruction (no reference object) and with proposed MLAA reconstruction including a bias correction based on known attenuation of a reference object. True emission and attenuation images are also shown as a reference

To test the proposed algorithm, multiple 2D simulations were performed. As input, reconstructed PET/CT images for a patient were extracted from an online database [28, 29] and one slice from the thoracic region was selected (see Fig. 1). The PET image used as true activity distribution has 128 × 128 pixels with a pixel size of 5 mm. CT image was resampled to the same voxel size as the PET image in order to obtain true attenuation images. Attenuation map was obtained by conversion from Hounsfield units (HU) to attenuation coefficients at 511 keV using the bilinear conversion proposed by Carney et al. [30] for the corresponding energy (130 kVp). True emission and attenuation sinograms were generated by forward projection of the true activity and attenuation images (see Eq. 2). Attenuated emission sinogram was generated as the product of both sinograms as follows:

6 $$ {y}_{i,\mathrm{TOF}}={b}_{i,\mathrm{TOF}}\cdotp {a}_i $$

The sinograms obtained had 90 angular samples over 180°, 256 radial samples with 2.5 mm bin size and 13, 27, or 81 TOF-bins for 540, 300, or 100 ps CRT, respectively.

Attenuated emission sinograms including noise were generated using an acceptance-rejection Monte Carlo (MC) method. For that purpose, a random sinogram entry (*i*,TOF) is chosen and a random number is generated between 0 and the maximum value of the noise-free sinogram. The event is added to the new sinogram if the random value is lower than the noise-free sinogram at entry (*i*,TOF). This process is repeated until the desired number of events is reached. In that way, a new sinogram is built with a predefined number of events distributed as the noise-free sinogram. The number of coincidences to be simulated was established by obtaining attenuated sinograms of a cylinder with 20-cm diameter filled with water and uniform activity with different number of coincidences. Those sinograms were reconstructed using standard MLEM algorithm with 100 iterations including known attenuation map. The standard deviation (SD) was computed in a ROI at the center of the reconstructed image and the number of events leading to a SD of 5% was selected as a reference for simulations with patient data which corresponded to 10^7^ coincidences for a CRT of 300 ps. In addition, simulations with lower number of coincidences (10^6^ and 10^5^) were also tested to evaluate the method at different noise levels.

In order to provide an initial estimate of the attenuation map for the MLAA algorithm, we performed a non-attenuation corrected MLEM reconstruction and the body contour was segmented using a Gaussian filter followed by a watershed algorithm. The attenuation coefficient within the patient volume was initialized as water. True attenuation values for the patient table and the reference object were included as a template in the initial attenuation map.

The proposed algorithm was tested with different configurations of the reference object including variations of the geometry, material composition, and activity. Initially, the reference object was defined as a water cylinder with 4-cm diameter inserted in the patient table with an activity concentration equal to the average activity concentration within the patient (*A*_0_). In addition, other activity values were tested including no activity, *A*_0_/4, and 4·*A*_0_. The geometry of the reference object was also tested including 2 and 4 water cylinders each one filled with *A*_0_/2 and *A*_0_/4, respectively, to preserve the total activity within the reference objects. For the case of 2 cylinders, an additional cylinder was placed on top of the patient and for the case of 4 cylinders, 2 more cylinders were added on both lateral sides of the patient. Finally, two other materials for the reference object were tested using one cylinder with *A*_0_ made of lung or bone equivalent materials with attenuation coefficients of 2.76·10^− 3^ and 12.01·10^− 3^ mm^− 1^, respectively.

A CRT of 300 ps was chosen to study all the configurations mentioned above which is similar to the TOF resolution of most recent PET scanners [31–33]. In addition, two other CRT values were studied to evaluate the performance of the proposed method in the previous generation of PET scanners with a CRT of 540 ps [34, 35] and in PET scanners with improved CRT (100 ps) that might be available in the future [36–38].

### Image analysis

The accuracy of the obtained reconstructed images was evaluated as follows. The patient volume was segmented into four tissue types (*t*) including lung, bone, soft tissue, and adipose tissue according to the attenuation coefficients included in the CT-derived attenuation map. The mean percentage difference (*∆*^*t*^) between the reconstructed (*x*_*j*_) and the true (${x}_j^{\mathrm{true}}$) emission images and the standard deviation of the percentage difference (SD^*t*^) were calculated for each tissue

7 $$ {\Delta }^t=\frac{1}{n^t}\sum \limits_j\frac{x_j-{x}_j^{\mathrm{true}}}{x_j^{\mathrm{true}}}\bullet 100 $$

where *n*^*t*^ is the number of pixels in the tissue *t*. In addition, pixel-wise maps with the percentage difference between the reconstructed and the true emission images were obtained.

## Results

First, the standard MLAA algorithm was applied without any reference object and therefore, Eqs. 4 and 5 were not applied (see Fig. 1). Reconstructed emission and attenuation images show a large deviation (> 50%) compared with true images due to the limitation mentioned by Defrise et al. [16]. The reconstructed images obtained with proposed method including a reference object and using the reference configuration (CRT = 300 ps, 10^7^ coincidences and reference object as one water cylinder with *A*_0_ activity) are also shown in Fig. 1 obtaining a much better agreement (mean differences are below 10% for all tissues).

**Fig. 2:**
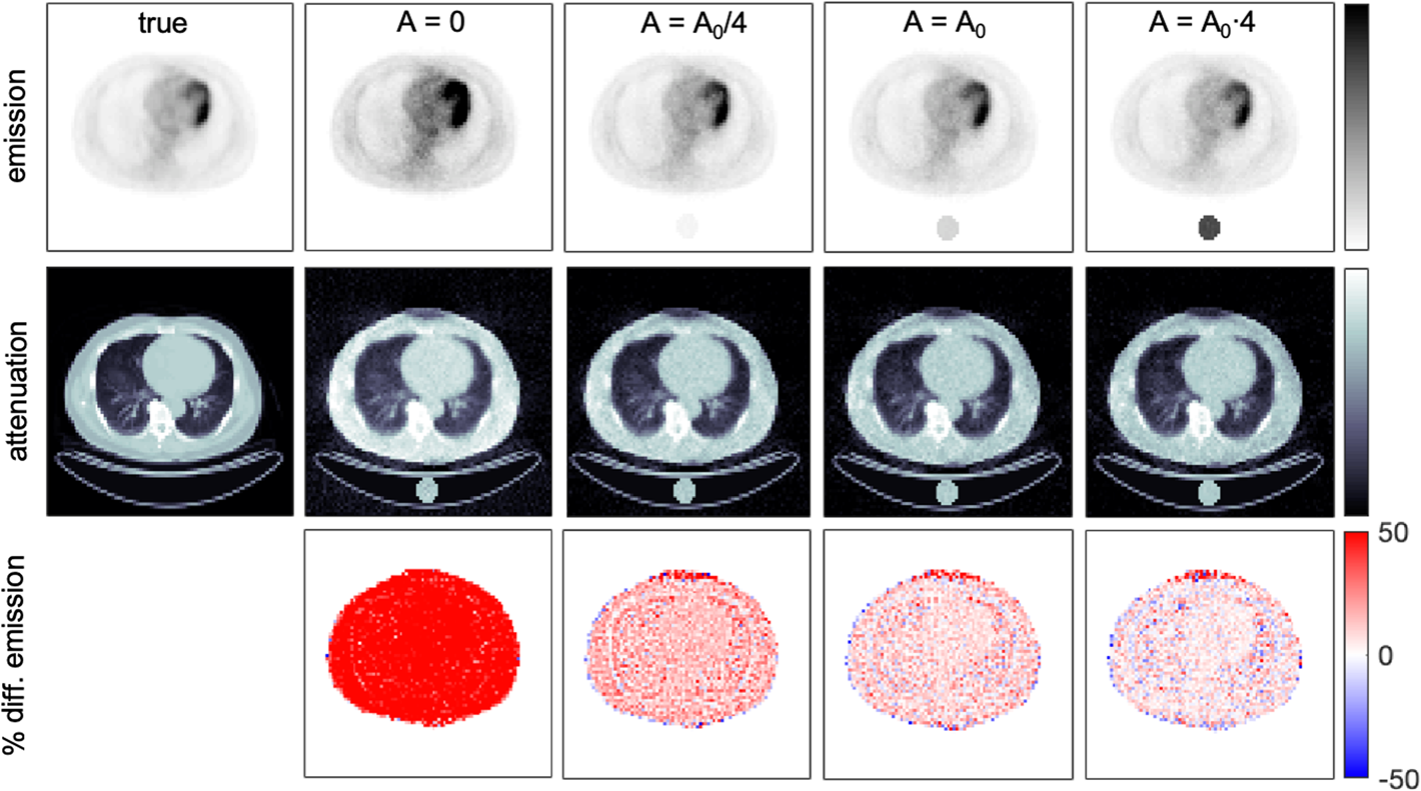
Reconstructed emission and attenuation images, percentage difference maps of emission images, and percentage difference maps of attenuation images obtained with proposed MLAA reconstruction using one water-filled cylinder with different activity concentrations (*A*). *A*_0_ is the average activity concentration within the patient. True emission and attenuation images are also shown as a reference

In order to study the influence of the activity concentration within the reference object, different cases were simulated and the resulting images are shown in Fig. 2. The bias of the reconstructed image increased when the activity was reduced while a similar SD was obtained for all cases when A > 0 (see Table 1). On the other hand, very large differences were obtained when no activity was placed in the reference object as the LORs with measured coincidences that cross the reference object are also crossing the patient and then, the limitation mentioned by Defrise et al. [16] remains. Therefore, some activity must be placed within the reference object to be able to implement this method. This activity should be high enough to improve convergence although a very high activity might deliver an unnecessary dose to the patient. Percentage difference maps of attenuation images are also shown in Fig. 2 where larger deviations can be observed at patient edges and lung interfaces with other tissues.

**Table 1:** Mean percentage difference between the reconstructed and the true emission images and SD for each tissue type obtained for all configurations included in this study. Reference configuration corresponds to proposed MLAA with reference object defined as one water cylinder (cyl) filled with *A*_0_ activity concentration, CRT = 300 ps and 10^7^ coincidences. Results obtained with fixed attenuation values in air pixels (F.A.) are included below

Configuration	Bone		Soft tissue		Adipose tissue		Lung	
	Mean	SD	Mean	SD	Mean	SD	Mean	SD
Standard MLAA	− 66.4	3.1	− 64.5	3.6	− 57.7	9.7	− 60.7	7.0
Reference	3.3	9.3	6.7	9.1	8.1	14.3	9.1	12.6
*A* = 0	54.4	14.0	61.0	13.4	59.6	21.5	64.8	19.4
*A* = *A*_0_/2	7.8	9.5	13.1	9.1	14.1	15.0	15.4	13.5
*A* = *A*_0_·2	− 0.8	9.4	3.2	8.4	4.5	13.2	5.5	13.5
Lung cyl	155.3	24.5	166.1	21.4	177.3	43.4	174.8	40.4
None cyl	− 1.9	8.8	3.8	8.6	5.3	13.5	5.7	12.8
2 water cyl	1.8	9.3	6.2	8.4	7.6	14.3	8.7	13.2
4 water cyl	1.9	9.5	6.2	9.1	8.6	14.3	8.9	13.7
100 ps, 10^5^	83.3	68.9	73.5	74.8	78.5	102.0	78.7	91.6
300 ps, 10^5^	104.2	153.0	120.2	148.4	124.5	194.1	126.7	195.0
540 ps, 10^5^	85.3	159.1	96.7	160.8	115.2	221.8	118.1	225.5
100 ps, 10^6^	15.3	14.9	17.2	16.1	17.9	21.8	17.4	19.9
300 ps, 10^6^	13.8	25.0	16.5	29.0	19.0	38.8	18.8	41.8
540 ps, 10^6^	40.7	48.6	51.2	47.6	57.1	67.7	61.5	75.9
100 ps, 10^7^	5.9	4.4	7.0	4.7	7.4	7.0	7.7	6.1
540 ps, 10^7^	− 2.6	15.9	8.4	13.2	11.9	19.5	16.2	22.2
Reference F.A.	7.0	9.0	8.4	10.7	7.5	20.5	9.8	14.4
Lung cyl F.A.	− 0.2	7.9	1.7	9.6	− 0.4	15.0	1.9	12.1
Bone cyl F.A.	9.6	9.0	10.6	10.4	10.0	21.8	11.7	14.2
A = 0 F.A.	15.2	9.9	17.7	11.2	13.9	23.0	18.7	15.9
A = A_0_/2 F.A.	16.8	9.0	18.4	11.3	15.5	22.6	19.5	16.9
A = A_0_·2 F.A.	− 1.7	8.9	0.0	9.3	− 0.4	17.6	1.3	14.2

*cyl* cylinder

**Fig. 3:**
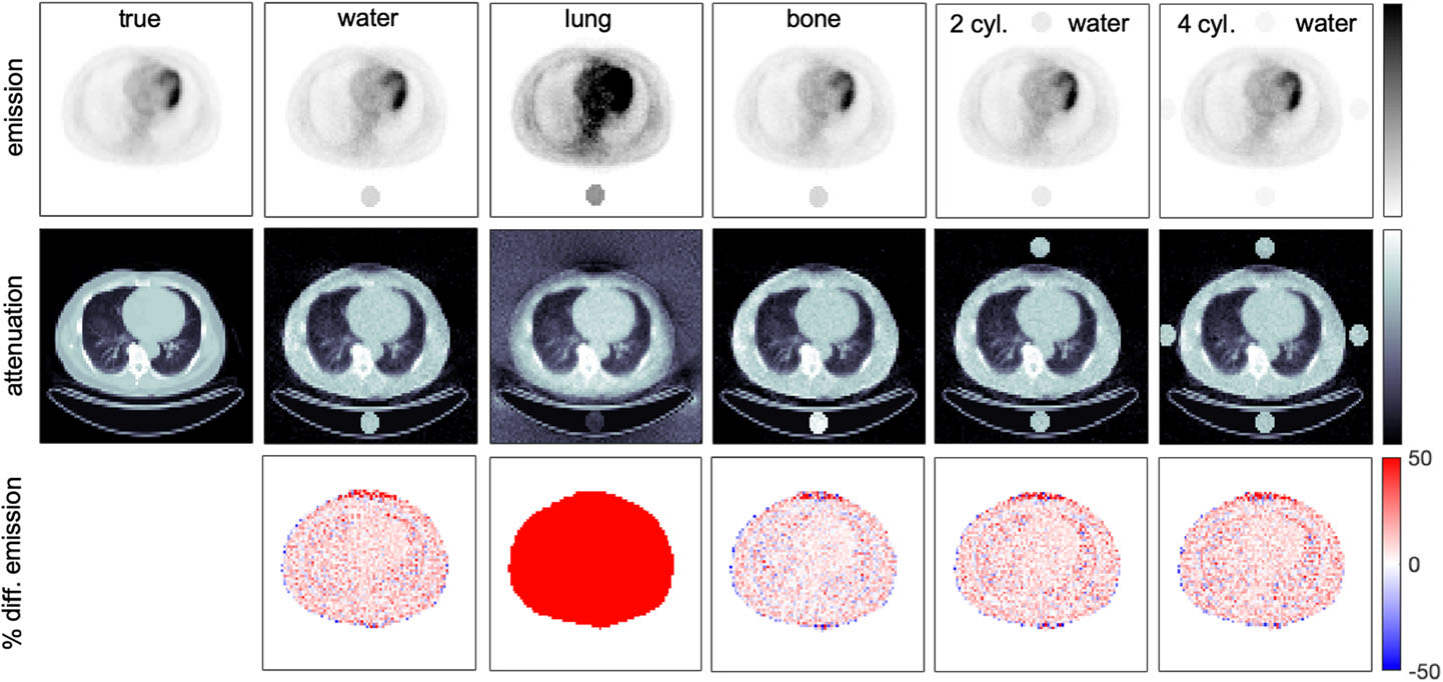
Reconstructed emission and attenuation images and percentage difference maps of emission images obtained with proposed MLAA reconstruction using one cylinder filled with reference activity concentration (*A*_0_) and materials with different attenuation coefficients (water, lung, and bone) and using 2 and 4 cylinders filled with water and the same total activity as for the one cylinder case. True emission and attenuation images are also shown as a reference

**Fig. 4:**
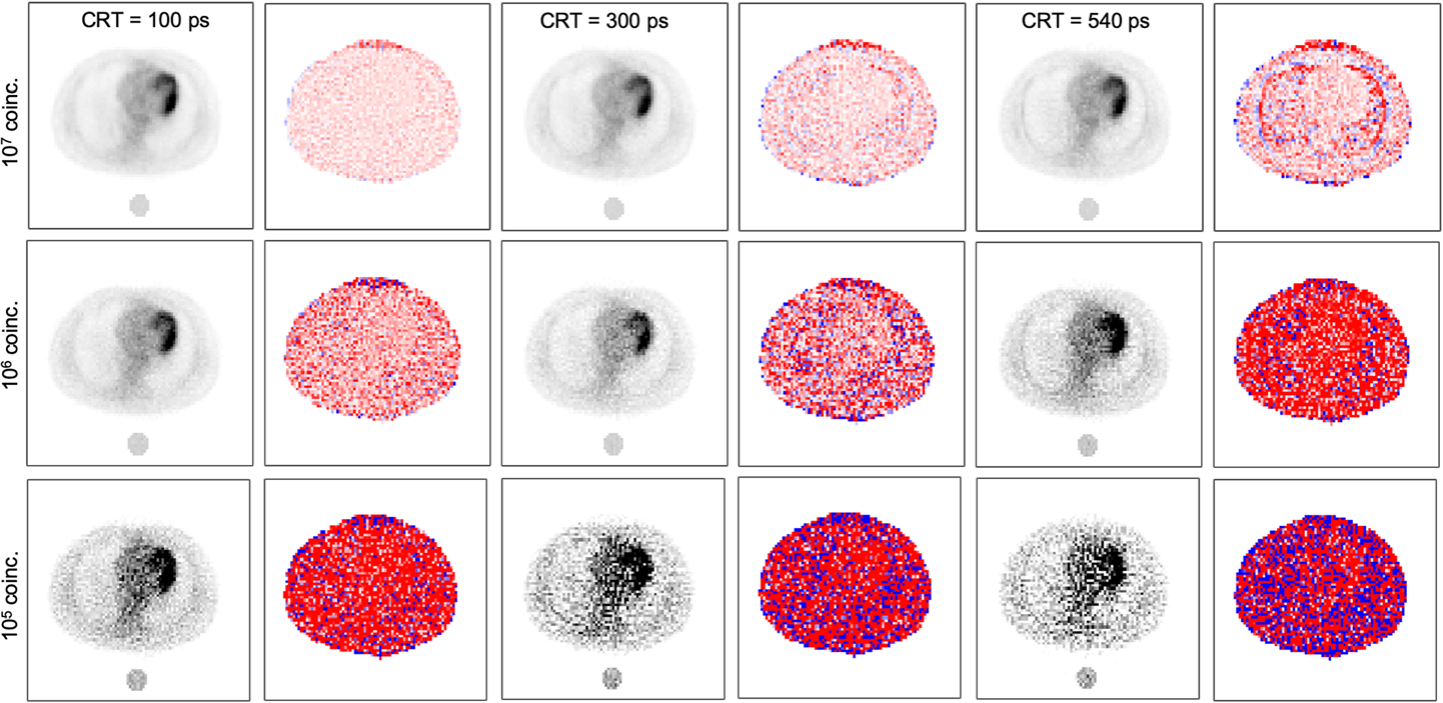
Reconstructed emission images and percentage difference maps of emission images obtained with proposed MLAA reconstruction using one water cylinder filled with reference activity concentration (*A*_0_). Results are shown for different CRT values (100, 300, and 540 ps) and different number of coincidences (10^5^, 10^6^, and 10^7^)

Other studied configuration parameters were the material and geometry of the reference object. Figure 3 shows the results obtained with one lung, water, or bone cylinder as well as with 2 and 4 water cylinders. Very large differences were obtained for the lung cylinder whereas all other cases show similar results as can be also observed in Table 1. Therefore, for simplicity, the case with one water cylinder and *A*_0_ activity concentration can be considered the best choice among tested options. This configuration of the reference object was also studied using different CRT and counting statistics and the results are shown in Fig. 4. As expected, the accuracy of the results improved with lower CRT and better results were obtained with a higher number of coincidences.

**Fig. 5:**
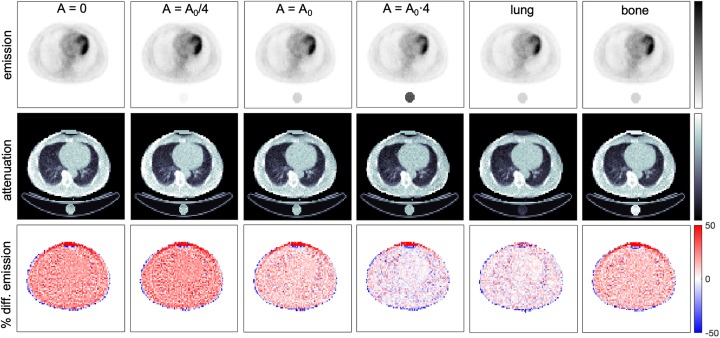
Reconstructed emission and attenuation images and percentage difference maps of emission images obtained with proposed MLAA reconstruction with fixed attenuation values in air pixels. Reconstructed cases include using as reference object one water-filled cylinder with different activity concentrations (*A*) and one cylinder filled with reference activity concentration (*A*_0_) and materials with different attenuation coefficients (lung and bone)

In all previous cases, the attenuation values were updated inside and outside the patient except for the patient table. In order to facilitate the convergence of the reconstruction process, we also tested some of the cases previously reported fixing the attenuation values outside the patient corresponding to air (see Fig. 5). Using this strategy, the lung equivalent material in the reference object provides good results while other cases with lower or no activity in the reference object still show large deviations.

**Fig. 6:**
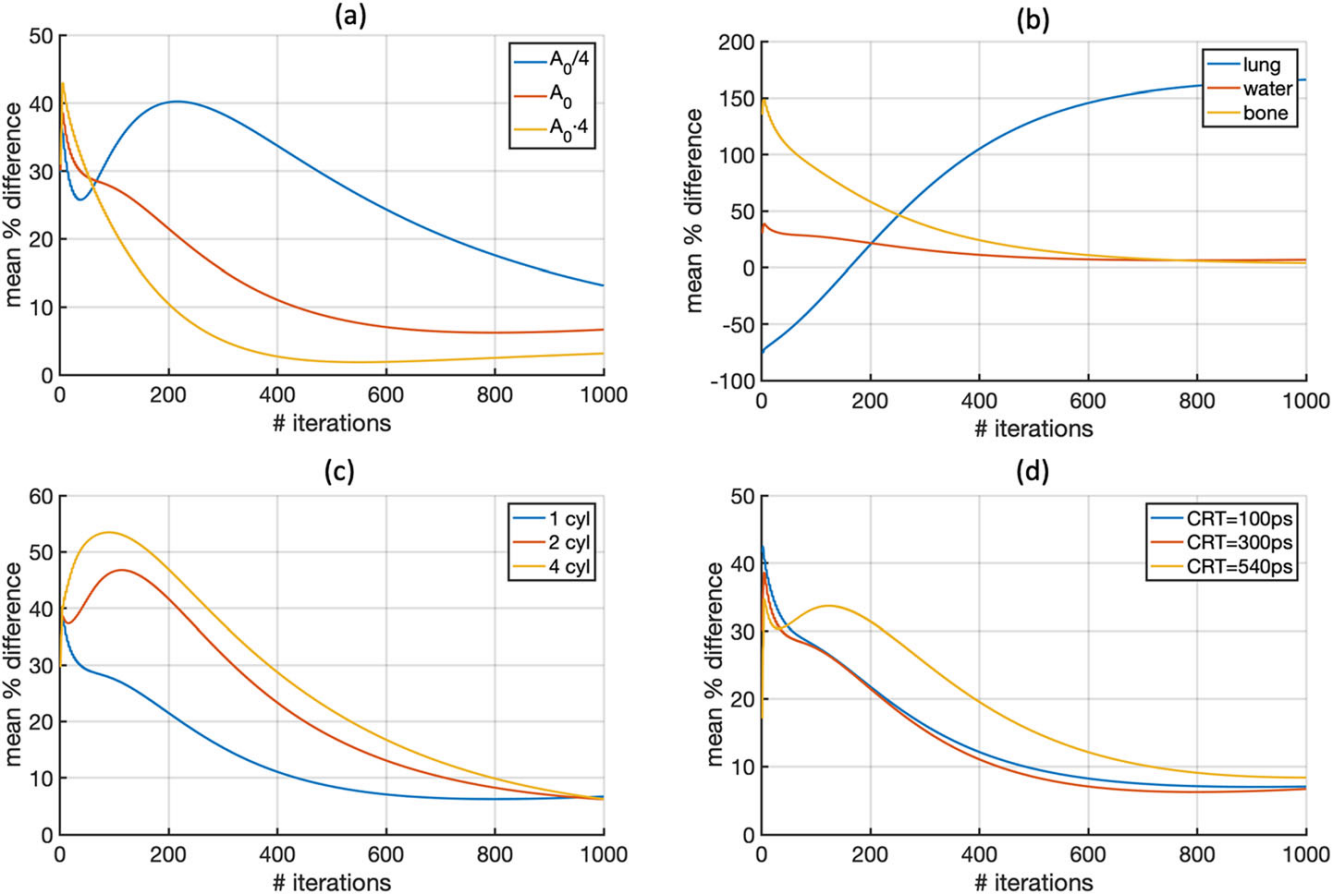
Mean percentage difference for soft tissue as a function of the number of iterations obtained for reference object with several activity concentrations (**a**), materials (**b**), and number of cylinders (**c**), and for different CRTs (**d**). All other options were set as in the reference configuration (CRT = 300 ps, 10^7^ coincidences and reference object as one water cylinder with *A*_0_ activity). All pixels of the attenuation image except for the patient table were updated during the reconstruction

Table 1 summarizes the mean percentage difference and the standard deviation obtained for all segmented tissue types on each of the configurations evaluated in this study. Finally, Fig. 6 shows the mean percentage difference obtained for soft tissue, as a function of the number of iterations, in order to compare the convergence of different configurations. The reliability and robustness of the proposed iterative method is illustrated in Fig. 6.

## Discussion

The simultaneous reconstruction of emission and attenuation images using a modified MLAA algorithm has been evaluated in this study trying to solve the fact that the attenuation sinogram can only be determined up to an additive constant with standard MLAA (see Fig. 1). As proposed by Defrise et al. [16], a reference object with known attenuation could be used to overcome this limitation but to the best of our knowledge no prior studies have been presented evaluating this option. In this study, we proposed a modification in the MLAA algorithm including a bias correction on the attenuation image based on the difference between the reconstructed values on the reference object and its known attenuation values. This strategy was tested in 2D simulations using different configurations of the reference object (material, activity, and geometry) using a CRT of 300 ps and 10^7^ coincidences in all cases. Obtained results suggest that the best configuration corresponds to one water cylinder filled with an activity concentration equal to the average activity within the patient although a higher activity would produce more accurate results and faster image convergence (see Fig. 5a) but delivering a higher dose to the patient. The addition of more cylinders around the patient produced minor differences after 1000 iterations although slower convergence was achieved (see Fig. 5c) and would require more complexity in the patient setup. Other geometries as full ring or larger cylinders were also studied (results not shown) but no improvements were obtained and those options were discarded due to the extra complexity of those setups [23, 39].

The chosen configuration for the reference object was tested with different CRT values and number of coincidences. For the high statistics case (10^7^ coincidences), mean percentage differences below 10% are shown in all cases except for adipose tissue and lung for a CRT of 540 ps. However, the larger differences obtained in lung and adipose tissue might be due to the fact that the activity concentration in the selected patient was much lower on those tissues than in bone and soft tissue and therefore, were more susceptible to noise variations. Hence, the proposed technique might be applicable in available TOF-PET scanners with both 300 and 540 ps.

When counting statistics become very low (10^5^ coincidences), as would be the case of short frames in dynamic studies, very large deviations were obtained for all CRT values. However, we must consider that the attenuation map could be obtained with the proposed method using a longer acquisition from the end of the dynamic study and later used in standard MLEM reconstruction for the dynamic data.

Further improvements could be included during the reconstruction process to avoid high noise levels and slow convergence of the algorithm [40]. However, the aim of this study was to evaluate the influence of the reference object under different conditions and further improvements of this method were left to future studies. In addition, this study was based on simple 2D simulations that did not include random or scatter events. Therefore, futures studies must evaluate this technique in complete 3D simulations and on measured data that should include phantom and patient data. Regarding patient data, other regions as the brain, prostate, or breast imaging should be also studied. One possible limitation of the proposed technique is its implementation in stand-alone PET scanners. Some of these scanners are very compact and there might not be enough room for a reference object. In that case, a more compact geometry of the reference object would be required.

## Conclusion

In this study, we tested a modified MLAA reconstruction algorithm that corrects the bias in the attenuation image using a reference object with known attenuation. Different configurations of the reference object were studied including geometry, material composition, and activity. An optimal configuration was defined. In addition, this configuration was tested using different CRT values in order to evaluate its performance in current and future PET scanners and under different noise levels to evaluate its accuracy in scenarios like low injected dose or dynamic PET scans. In conclusion, this study shows promising results that might allow to obtain accurate attenuation map from pure TOF-PET data without prior knowledge from CT, MR, or transmission scans. However, further studies are needed to test this method under more realistic conditions.

## Data Availability

Data and materials are available on request to the authors.

## Abbreviations

CRT: Coincidence Resolving Time
CT: Computed Tomography
HU: Hounsfield Units
LOR: Line of response
MLAA: Maximum likelihood estimation of attenuation and activity
MLEM: Maximum likelihood expectation maximization
MR: Magnetic resonance
PET: Positron emission tomography
ROI: Region of interest
SD: Standard deviation
TOF: Time of flight
